# Cross Attention Squeeze Excitation Network (CASE-Net) for Whole Body Fetal MRI Segmentation

**DOI:** 10.3390/s21134490

**Published:** 2021-06-30

**Authors:** Justin Lo, Saiee Nithiyanantham, Jillian Cardinell, Dylan Young, Sherwin Cho, Abirami Kirubarajan, Matthias W. Wagner, Roxana Azma, Steven Miller, Mike Seed, Birgit Ertl-Wagner, Dafna Sussman

**Affiliations:** 1 Department of Electrical, Computer and Biomedical Engineering, Faculty of Engineering and Architectural Science, Ryerson University, Toronto, ON M5B 2K3, Canada; justin1.lo@ryerson.ca (J.L.); foustina.nithiyanant@ryerson.ca (S.N.); jillian.cardinell@ryerson.ca (J.C.); dylan.young@ryerson.ca (D.Y.); 2 Institute for Biomedical Engineering, Science and Technology (iBEST), Ryerson University, St. Michael’s Hospital, Toronto, ON M5B 2K3, Canada; sherwin.cho@mail.utoronto.ca; 3 Faculty of Medicine, University of Toronto, Toronto, ON M5S 1A8, Canada; abi.kirubarajan@mail.utoronto.ca; 4 Division of Neuroradiology, The Hospital for Sick Children, Toronto, ON M5G 1X8, Canada; matthias.wagner@sickkids.ca (M.W.W.); roxanaazma@gmail.com (R.A.); birgitbetina.ertl-wagner@sickkids.ca (B.E.-W.); 5 Division of Neurology, The Hospital for Sick Children, Toronto, ON M5G 1X8, Canada; steven.miller@sickkids.ca; 6 Division of Cardiology, The Hospital for Sick Children, Toronto, ON M5G 1X8, Canada; mike.seed@sickkids.ca; 7 The Keenan Research Centre for Biomedical Science, St. Michael’s Hospital, Toronto, ON M5B 1W8, Canada; 8 Department of Obstetrics and Gynecology, Faculty of Medicine, University of Toronto, Toronto, ON M5S 1A8, Canada

**Keywords:** deep learning, fetal MRI, convolutional neural networks, automatic segmentation, squeeze-and-excitation, attention mechanisms

## Abstract

Segmentation of the fetus from 2-dimensional (2D) magnetic resonance imaging (MRI) can aid radiologists with clinical decision making for disease diagnosis. Machine learning can facilitate this process of automatic segmentation, making diagnosis more accurate and user independent. We propose a deep learning (DL) framework for 2D fetal MRI segmentation using a Cross Attention Squeeze Excitation Network (CASE-Net) for research and clinical applications. CASE-Net is an end-to-end segmentation architecture with relevant modules that are evidence based. The goal of CASE-Net is to emphasize localization of contextual information that is relevant in biomedical segmentation, by combining attention mechanisms with squeeze-and-excitation (SE) blocks. This is a retrospective study with 34 patients. Our experiments have shown that our proposed CASE-Net achieved the highest segmentation Dice score of 87.36%, outperforming other competitive segmentation architectures.

## 1. Introduction

MRI plays a key role in fetal diagnosis due to its high resolution, superb soft-tissue contrast, and 2D capabilities [1]. Automatic segmentation of the whole fetus provides valuable insights into fetal development and serves as an important tool for advanced diagnostics. Accurate volumetric segmentation of the entire fetal body facilitates understanding of fetal growth rate, early diagnosis of pathologies, and improved surgical planning [2]. Rapid development in DL over the past decade has given rise to numerous techniques suitable for automatic fetal image analysis.

DL employs convolutional neural networks (CNN) to perform an in-depth analysis of images and multidimensional data forms. These neural networks use a complex series of convolutions, maximum pooling, and up-sampling operations to learn advanced image analysis tasks efficiently and accurately. Previous studies have found that DL algorithms are robust and accurate. They can reduce the time and cost required for segmentation and diagnostic tasks [3]. This makes DL an ideal tool for automated whole fetal segmentation, due to the diversity in both morphological and image quality in conventional fetal imaging.

Segmentation algorithms have a variety of clinical applications ranging from cardiac segmentation to infant brain segmentation [4]. DL algorithms contain many variables and functions that can be modified to optimize results, leaving room for improvement in any algorithm. The U-Net architecture is a popular choice of CNN, specifically for medical image segmentation applications. U-Net is unique in that the model performs up-sampling (i.e., interpolation) after convolution instead of the traditional pooling operation. This allows the algorithm to recognize local features and reassess the important details within an image [5]. U-Net also performs concatenation operations on the output layer of the decoder (i.e., the contracting path) and its parallel up-sampling/encoding (i.e., expanding path) layer. This design creates symmetry in the U-Net model, as the down-sampling layers are being matched to the up-sampling layers. Concatenated data are then synthesized to develop more precise segmentation [5].

DL has been a popular choice for a variety of fetal imaging applications. Previous studies have described accurate DL algorithms for brain or brain tumor segmentation for fetal MRI [6,7]. Existing DL-based fetal segmentation algorithms include: a VGG16-based encoder/decoder type network to segment fetal tissue and amniotic fluid from ultrasound images [8]; a multiscale CNN on 2D slices of fetal MRI to segment intracranial volumes [9]; a combination of a CNN with data augmentation to segment intracranial volume and seven brain tissue types [10]; two CNNs for coarse and fine segmentation and fetal brain reconstruction from MRIs [11]; dynamic CNNs applied to echocardiography to segment the left fetal ventricle which, when combined with gradient boosting machines, was used to segment the fetal abdomen in ultrasound images [12]; a CNN for fetal skull segmentation from 3D ultrasound [13]; and a semi-automated bounding box with a CNN pipeline for segmenting maternal-fetal organs from 2D MRI slices [14].

In standard CNNs, including U-Net, the weights of each channel all have the same value. The squeeze-and-excitation (SE) block adds adaptive weights to the channel-wise feature maps by modelling the interdependencies between these convolutional channels [15]. This implemented mechanism allows for feature recalibration using global information to determine which features are emphasized, and which are minimized. The SE block achieves this by squeezing each channel to a single value by using global average pooling. This value then undergoes a series of operations including a fully connected layer, a Rectified Linear Unit (ReLU), and a sigmoid activation function, resulting in the desired nonlinear characteristics. These nonlinear features are mapped back to the original channel, allowing all feature maps to obtain adaptive weights. The SE block offers convenient implementation to any end-to-end network and accounts for only a minor increase of less than 0.5% in computational cost [16].

The addition of attention mechanisms before the concatenation step can provide greater emphasis on contextual information, which is likely to lead to higher segmentation accuracy. Skip connections can combine features from the contracting pathway into the expanding pathway by concatenating the two. The attention mechanism then combines the two signals from the skip connection and from the previously deconvoluted layer, in order to determine the significance of relevant features [17,18,19]. The result is an attention map that is multiplied by the input to the skip connection to produce the attention block. As such, we hypothesized that there would be synergistic effects between applying adaptive weights to feature channels in the contracting pathway, as well as further emphasis on these weighted features. We anticipated that a network incorporating these features would have improved segmentation results.

**Figure 1:**
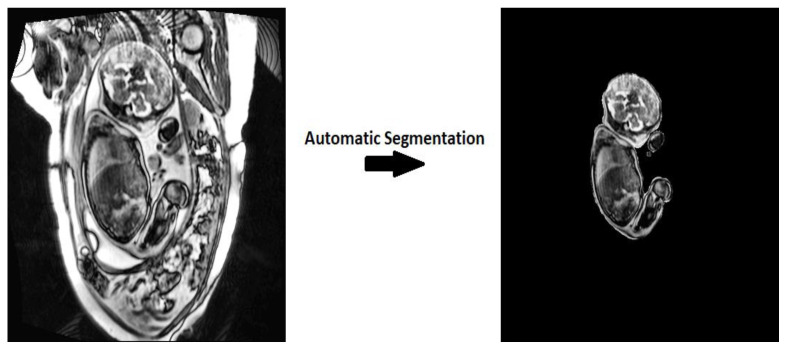
The given MRI dataset is applied through an automatic segmentation algorithm for clinical diagnosis.

In the current study, we take advantage of the benefits offered by DL to develop a new architecture that can automatically and accurately segment the whole fetal volume from the respective MRI slice, using the U-Net architecture as the primitive framework. Given the success of U-Net in previous applications, we hypothesized that our proposed architecture would also perform as an efficient and useful segmentation tool that can improve the evaluation of fetal health and development. This work aims to develop a novel architecture by using previously established building blocks from SE blocks, attention mechanisms, and hyper-parametric changes. Our proposed architecture aims to contribute to improvements in clinical settings and provide a novel architecture for biomedical image segmentation. Figure 1 illustrates the aim of our proposed work.

The contributions of this work are as follows:

1. Curation of a manually segmented sagittal fetal MRI dataset;
2. Design of a novel CNN-based architecture for the automatic segmentation of fetal MRI images, with comparable results to state-of-the-art methods.

Section 2 of this paper covers the research methodology, data used, algorithm architecture, hyper parametric changes, experiments, and statistical analyses. Section 3 then covers the experimental results and differences between architectures. Section 4 provides a discussion on the interpretation of the results and the significance of the findings. Finally, Section 5 summarizes the conclusion and key findings of this paper.

## 2. Materials and Methods

### 2.1. Acquisition

De-identified patient data of whole-body fetal MRIs were acquired using various sequences, as part of several previous studies (The Hospital for Sick Children, Toronto, Canada). Namely, an SSFP sequence was used on a 1.5 T scanner and a 3D SSFP with SENSE along two dimensions (locally referred to as a CHOP sequence [20]) was used on a 3.0 T scanner. Sagittal images were used as this view provides the most surface area for fetal anatomy. Only one scan per patient was used if multiple ones were available. The dataset was composed of normally developing fetuses with no evident pathologies. Exclusion criteria consisted of severe artifacts and pathologically unhealthy fetuses. Minor artifacts were not excluded, as they contained very minimal motion, chemical shift, or radiofrequency distortion. As such, these minor artifacts did not significantly degrade image quality and were, therefore, included in the dataset. Gestational age at the time of acquisition was between 20 and 37 gestational weeks (gw) with a distribution centered at 28 gw (28.42 ± 4.62 gw; mean ± std). Gestational age was evenly distributed across the training, validation, and testing datasets to avoid bias in any one dataset.

### 2.2. Dataset

This retrospective study was approved by the local research ethics board and requirement for informed consent was waived due the retrospective nature. Segmentation was conducted by one of the three independent observers who were all equally trained on identifying fetal anatomy in 2D MRIs. We used the Amira-Avizo software (Thermo Fisher Scientific, Berlin, Germany) to manually segment the fetuses. The data were divided into training, validation, and testing datasets in a 60/20/20 split. There were 4032 total images; 2368 of which were used in the training set while 832 images were used for both the testing and validation. Thirty-four T2-weighted sagittal fetal MRI datasets were used to generate these images, each containing 119–120 slices. Image processing was performed on the dataset in the form of intensity normalization to create a similar distribution amongst our data. This was necessary as our data consisted of images that were acquired using different image sequences (SSFP and CHOP) and parameters, resulting in a large range of intensity values.

### 2.3. CASE-Net Architecture

**Figure 2:**
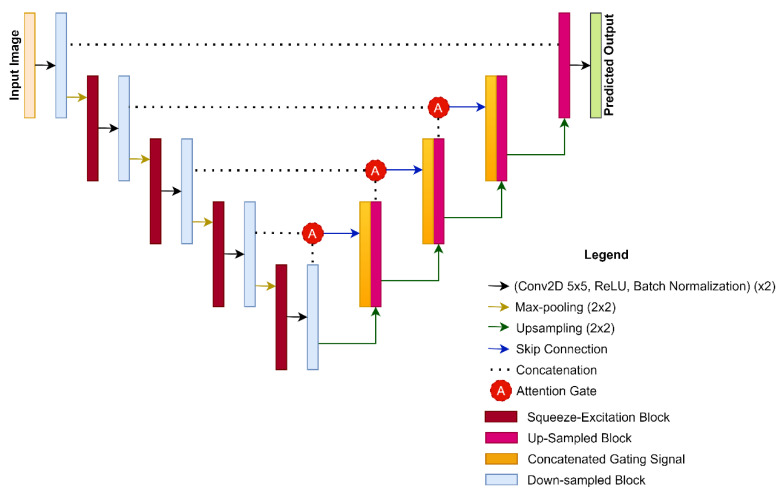
An overview of the proposed CASE-Net architecture.

The proposed architecture is illustrated in Figure 2 using the U-Net architecture as the framework. The encoding pathway consists of four SE blocks applied after every max-pooling operation. An attention gate was added after every deconvolution operation that becomes concatenated with the excited channels from the SE block from the encoding pathway. This further emphasized the relevant features that are useful for whole fetal MRI segmentation. SE blocks and attention gates were not added in the superficial convolutional layer due to the lack of relevant contextual information as well as computational constraints.

### 2.4. Squeeze-and-Excitation Networks

**Figure 3:**
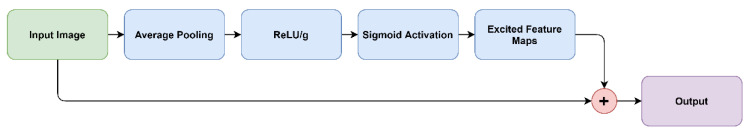
Block diagram of the SE block.

Implementing SE blocks directly after the max-pooling operation in the encoding pathway allows the previous convolutional layers to have adaptive weights for its feature channels. This leads to the emphasis of important features, as layers from the encoder are concatenated with the attention modules. Figure 3 illustrates the process of implementing the SE block in the end-to-end network.

SE blocks consolidate the input feature maps and squeeze the feature channels using global average pooling to reduce the input to a single value across all channels. Then the model achieves the desired non-linearity by going through ReLU activation, divided by a complexity value g = 8. The complexity value reduces the parameters in the output channel. A complexity value of 8 is typically used in networks that implement SE blocks [15]. The side path of the input is then multiplied by the excited maps, leading to feature maps with adaptive weights [15,16].

(1) $$Z_{n}=F_{squeeze}\;\left(U_{n}\right)=\frac{1}{H\cdot W}\;\overset{H}{\underset{h=1}{\sum }}\overset{W}{\underset{w=1}{\sum }}U_{n}\;\left(i,j\right)$$

The squeezing operation can be described in (1) by $F_{squeeze}()$. Where $U_{n}$ represents the corresponding feature map, where the size of a channel $U_{n}$ is modelled by the spatial dimensions H×W. By using global average pooling, we produce an average value representing each channel such that $Z_{n}$ represents the *n-th* element.

(2) $$s=\;F_{excite}\;\left(Z_{n}\;\right)=\left(\sigma ,\;\left(\delta ,\frac{Z_{n}}{g}\right)\right)$$

The excitation operation can be described in (2) as $F_{excite}()$. Modelling the interdependencies between the channels requires a fully connected layer,

ReLU activation, and sigmoid activation, where σ is the sigmoid activation function, δ is the ReLU activation, *g* is the reduction of channel complexity. The channel is then fed through the two activations to achieve nonlinearity. $Z_{n}$ is divided by the complexity value g and applied to the δ ReLU activation function, then finally it is applied to the σ sigmoid activation function.

(3) $$\underline{X}=s_{n}u_{n}\;$$

The last operation requires reshaping the output *U* in order to apply the activations to the side path network, where $\underline{\;X}=\left[\;x_{1},\;x_{2},\ldots ,\;x_{n}\right]$ and $s_{n}u_{n}$ refers to the channel wise multiplication between the scalar $s_{n}$ and the feature map. This process provides the adaptive weights to the feature channels, which is the premise of the SE block [16].

### 2.5. Attention Modules

Standard U-Net architectures use multilevel cascaded convolutional layers to make dense predictions [17]. However, this can lead to redundancy and computational inefficiency. Attention mechanisms in unison with SE blocks and U-Nets enable object localization that can suppress irrelevant features and enhance relevant features.

Both inputs from the encoder and the gating signal are fed through a 1 × 1 convolution, to ensure consistency in the number of channels while maintaining their original size. The results from both paths are summed and passed through a ReLU function, another 1 × 1 convolution, and finally through a sigmoid activation. The result is a distribution from 0 to 1 of the importance of the corresponding feature channel, in which higher values correspond to higher importance. The product is an attention map that is then multiplied by the skip connection from the gating signal to produce the attention block, where further deconvolution takes place. This concept is similar to SE blocks where a map is created, which is then applied to the input to improve adaptivity [18,19].

(4) $$a=F_{attention\;}\left(\;\underline{X},\;g_{s}\right)$$

**Figure 4:**
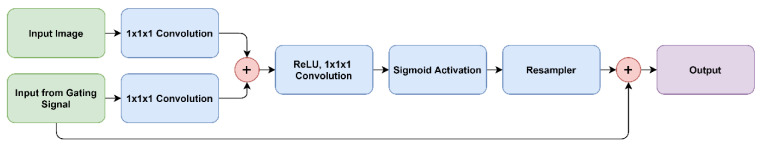
Block diagram of the Attention Mechanism.

The output $\;\underline{X}$ from the SE block is then used as one of the inputs for the attention mechanisms along with $g_{s}$, the gating signal from the current deconvolution. This is illustrated in Figure 4.

### 2.6. Hyper Parametric Changes

Our proposed architecture used a convolutional kernel of 5 × 5 as opposed to the standard 3 × 3 size. A larger kernel size has a larger receptive field that is likely able to generalize and identify superficial features (e.g., important edges). Another option is to stack two 3 × 3 convolutional kernels in a series. However, this method is limited by computational constraints, and may lead to overfitting. The same concept can be applied to stacking two 5 × 5 convolutional kernels to obtain an ever-greater receptive field. However, we stopped at 5 × 5 convolutional kernels as further increases would require much more computational resources. In typical biomedical imaging applications of CNNs, standard deconvolution in the form of up-sampling is used. In our architecture, we substituted up-sampling with a transposed convolution. Standard up-sampling has a predefined interpolation method which can be improved upon with learnable weights. These weights were designed to better enhance the up-sampled images [21].

### 2.7. Experiments

The proposed method was trained and tested on an Nvidia RTX 2070 Super graphics card with an 8 GB GDDR6 video memory, 2560 stream processors, 1605 MHz base clock, and a 1770 MHz boost clock. The model was programmed in Python using Keras with TensorFlow as the backend. The Adam optimizer was used in place of the standard stochastic gradient descent (SGD). This optimization algorithm has adaptive learning rates per-parameter as opposed to a singular learning rate in SGD. This optimization approach achieves better performance in CNNs with sparse gradients and non-stationary problems. The initial learning rate was set to 1 × 10^−4^. A learning rate scheduler was set to 1 × 10^−5^ if the performance of the network remained stagnant. Binary cross-entropy was used for our loss function. The size of the input images was 384 × 384 pixels. The batch size was set to 4, and 100 epochs were used for training. CASE-Net, U-Net, USE-Net, ATN-Net, and Link-Net were created manually in Keras using a sequential model (for details about the corresponding architectures, see [5,16,19,22]). We chose to compare our architecture with U-Net, USE-Net, ATN-Net, and Link-Net as these prominent 2D segmentation architectures are frequently used in medical image segmentation and result in high accuracy [5,16,19,22]. USE-Net and ATN-Net use SE blocks and attention mechanisms, respectively, enabling us to better understand the relative contributions of each of these modules to the overall performance of our architecture.

A 5-fold cross validation was used during training to create a more robust data set. A basic set of data augmentation transformations was applied on the data. This set of basic transformations included: horizontal stretch, vertical stretch, shear, zooming, and horizontal flip.

Three metrics were implemented to evaluate the segmentation accuracy: Dice Similarity Coefficient (DSC), recall, and precision. DSC is given by the following equation, which was calculated per 2D slice:

(5) $$\mathrm{DSC}=\frac{2\left|X\;\cap \;Y\right|}{\left|X\right|+\left|Y\right|}=\frac{2TP}{2TP+FP+FN}$$

Here, *X* and *Y* represent the cardinalities of two unique images. The DSC can be interpreted as twice the number of elements that are in union with each other, divided by the total sum of the elements. This equation can also be represented as variables in a confusion matrix, where *TP*, *FP*, and *FN* are the true positive, false positive, and false negative values, respectively. This equation is widely used to measure the similarity between two objects and was used as the primary decision metric for our research.

(6) $$\mathrm{Recall}=\frac{TP}{TP+FN}$$

(7) $$\mathrm{Precision}=\frac{TP}{TP+FP}$$

Recall, which is also referred to as the true positive rate or sensitivity, is the percentage of total relevant results that are correctly classified by the algorithm. Precision is the percentage of positive identifications that were correct by the algorithm. Recall and precision were the secondary metrics used to evaluate the performance of our algorithm, since DSC already incorporates both false negative and positive values, therefore, acting as a mean of these two metrics.

### 2.8. Statistical Analyses

Architecture performance was evaluated by using an independent *t*-test that was calculated on the DSC, recall, and precision. However, emphasis is placed on *p*-values comparing the DSC, as the other metrics are not as useful when trying to evaluate performance. A 95% confidence interval and a significance level of *p* < 0.05 was used.

Intra-observer agreement was analyzed by calculating the mean-squared error (MSE) for CASE-Net’s testing labels between both a clinician’s and a non-clinician’s manual segmentations. An independent *t*-test was used to compare the datasets. Table 1 depicts the statistical analysis of the segmentation. A 95% confidence interval was used, and we obtained a *p*-value of 0.63. This demonstrates that there were no significant differences between the individual segmentations, confirming intra-observer agreement.

## 3. Results

The segmentation performances of CASE-Net and the other competitive architectures are shown in Table 2. CASE-Net achieved a DSC of 87.36% which was significantly higher than the DSC of UNet, USE-Net, ATN-Net, and LinkNet by 2.19% (*p* = 0.048), 1.29% (*p* = 0.034), 5.09% (*p* = 0.019), and 4.64% (*p* = 0.021), respectively. With a 91.79% recall, CASE-Net also outperformed the other architectures by 2.55% (*p* = 0.022), 1.29% (*p* = 0.059), 5.09% (*p* = 0.078), and 4.64% (*p* = 0.058). Nonetheless, CASE-Net achieved a moderate 95.54% precision score, comparable to the other architectures.

The segmentation performances were also calculated in an ablation study for CASE-Net, as shown in Table 3. CASE-Net achieved a DSC of 87.36% which was significantly higher than the DSC of a 3 × 3 Kernel CASE-Net, without attention mechanisms and SE blocks by 3.86% (*p* = 0.0257), 3.01% (*p* = 0.0304), and 6.17% (*p* = 0.0331), respectively. CASE-Net also achieved the lowest loss of 0.005, which was lower by 0.047 (*p* = 0.1581), 0.126 (*p* = 0.0425), and 0.091 (*p* = 0.0138). With a 91.79% recall, CASE-Net also outperformed the other networks by 5.22% (*p* = 0.0455), 2.64% (*p* = 0.0750), and 10.84% (*p* = 0.0421). CASE-Net, however, had a relatively low precision score of 0.93% (*p* = 0.0118), 0.72% (*p* = 0.0498), and 1.17% (*p* = 0.0385), respectively.

**Figure 5:**
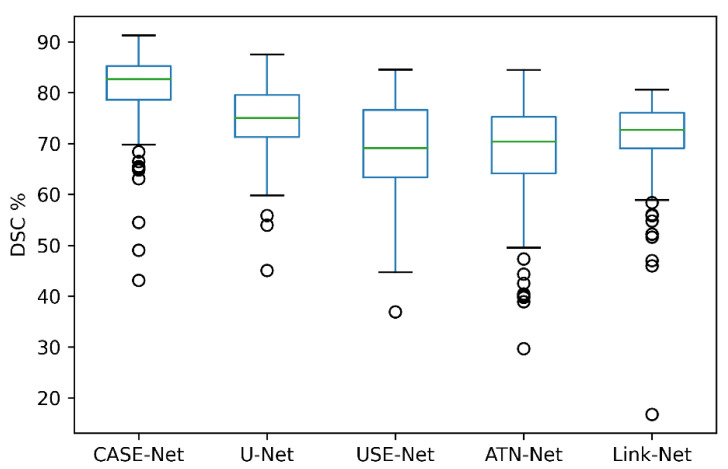
Boxplot representation of the DSC on various segmentation architectures.

Figure 5 visually depicts the segmentation performance of our proposed CASE-Net and the other competitive architectures. As shown, CASE-Net had the highest DSC when compared to U-Net, USE-Net, ATN-Net, and Link-Net. Relative to the other networks, CASE-Net demonstrated the highest $Q_{0}$ and $Q_{4}$ values. All networks were calibrated using their optimal loss, optimizers, and learning rates from their respective papers. In addition, each network used the same 5-fold validation, batch size, number of epochs, image augmentations, and dataset.

## 4. Discussion

Our study presents a novel CNN architecture, CASE-Net, which can achieve accurate 2D whole body fetal MRI segmentations when trained on second and third trimester images. The results show that CASE-Net outperforms other prominent architectures. Specifically, the CASE-Net architecture obtained the highest DSC of 87.36% compared to U-Net, USE-Net, ATN-Net, and Link-Net. These results indicate that a combination of attention mechanisms, SE blocks, and hyper-parametric changes can increase automatic segmentation performance. In addition, CASE-Net also demonstrated the highest recall score and moderate precision scores, indicating a low rate of false negatives and a propensity for false positives. The high recall score and low precision score demonstrates that CASE-Net tends to over-segment images.

Though U-Net is the most popular architecture for biomedical segmentation, the model demonstrated lower DSC, recall, and precision scores when compared to our proposed CASE-Net. However, USE-Net, ATN-Net, and Link-Net architectures demonstrated slightly higher precision but lower recall and DSC scores, indicating that these architectures under-segment images.

We conducted our ablation study (outlined in Table 3) to determine which individual components were responsible for the relative success of CASE-Net. The first experiment demonstrated that the 3 × 3 kernels had lower performance in all metrics except precision. The second experiment removed the attention mechanism from the network, showing a smaller impact in comparison to changing the kernel sizes. Despite the lack of attention mechanism, this architecture demonstrated lower results in DSC and recall, but higher precision. The last experiment did not use SE blocks, which demonstrated the greatest impact on performance, as the DSC decreased to 81.19%. Similar to the previous two experiments, the precision of the model remained at a higher value than CASE-Net. It can be seen that all of the networks, other than CASE-Net, tend to under-segment images. However, when all modules are combined, they achieve improved performance.

Embedding attention mechanisms in the U-Net architecture does not seem to produce desirable results in fetal MRI segmentation applications. However, when paired with SE blocks and integrated into the skip connections, this results in increased emphasis on the features received from the contracting pathway. The low accuracy obtained from the basic ATN-Net may be attributed to the nature of medical images, as the variation amongst slices may create challenges in emphasizing important features.

Our results show that SE blocks lead to improvement in segmentation performance when added to a DL architecture. Since many architectures do not implement adaptive weights to their model, localization of important features is not easily determined, thus leading to weaker performance. USE-Net has a low recall score despite having a high precision score, demonstrating that the model carefully identifies correct segmentations, although sometimes misses pixels. The discrepancy between recall and precision can be attributed to the SE blocks adding weights to important features, as identified by the algorithm.

**Figure 6:**
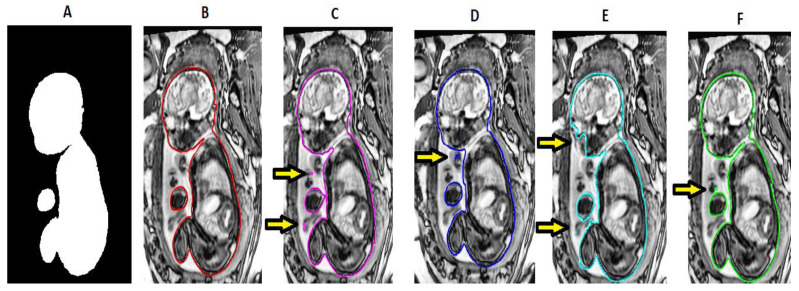
Predicted segmentation outputs of various architectures. (**A**): Label, (**B**): CASE-Net, (**C**): U-Net, (**D**): USE-Net, (**E**): ATN-Net, (**F**): Link-Net.

**Figure 7:**
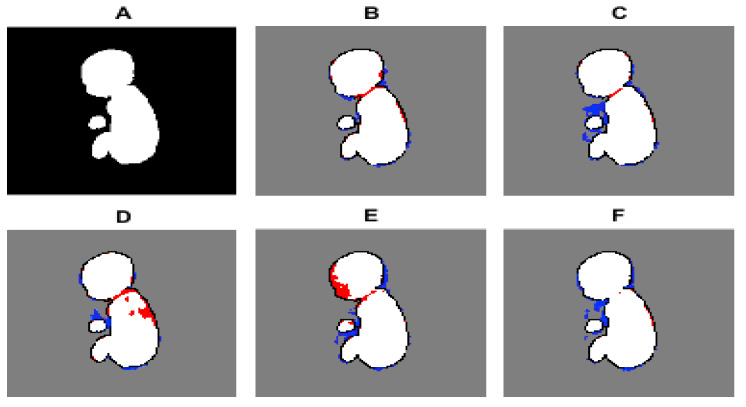
Pixel-to-pixel comparison of the ground truth mask against the predicted segmentations of various architectures. (**A**): Label, (**B**): CASE-Net, (**C**): U-Net, (**D**): USE-Net, (**E**): ATN-Net, (**F**): Link-Net. TP is given by the color white, TN is given by the color grey, FP is given by the color blue, and FN is given by the color red.

Figure 6 illustrates the predicted segmentation of the various architectures that were used. It can be seen qualitatively that U-Net and USE-Net tend to under segment the image around the fetal neck and spinal cord. ATN-Net and LinkNet under-segment around the fetal head and incorrectly segment the umbilical cord. On the other hand, CASE-Net produces a good balance between over- and under-segmentations and can segment the whole fetal MRI with a moderate degree of accuracy. In the studied architectures, gestational age did not have any effect on segmentation accuracy. Figure 7 illustrates the pixel-to-pixel comparison of the ground truth with the predicted segmentations’ masks of the various architectures. It can be seen that CASE-Net has very few FP and FN values compared to the other architectures.

Previous segmentation studies have focused heavily on frameworks using a modified U-Net architecture. These studies tend to obtain DSC values ranging from 84–92%, depending on the application [16,17,18,19,20,21,22]. Our study obtained DSC values between 87% and 89%, which are comparable. Yet, higher DSC results (i.e., over 90%) were not attained. The slightly lower DSC values in our study are likely due to the increased heterogeneity of the fetal body position and size compared to other biomedical imaging datasets. This leads to increased difficulty in segmentation, resulting in the slightly lower segmentation accuracy presented in this study [23]. When using our segmented fetal MRI dataset in other segmentation architectures, we did not find a model that yielded higher results than our proposed architecture. We speculate that the lack of emphasis on contextual information in other networks is the reason for our more accurate results.

Our results demonstrated that CASE-Net is a successful architecture that yields promising results. Further improvements to the model would require more computational power. It is likely that concatenating shallow layers with attention gates and SE blocks will further improve segmentation performance, but we were unable to achieve this in our proposed architecture due to limited computational resources. CASE-Net may also benefit from using advanced data augmentation techniques. The added variability would likely improve robustness to translations, rotations, intensity changes, and other forms of variations that occur naturally.

The advantages of the proposed architecture seem to demonstrate that the model performs well with biomedical datasets. The architecture is proficient in addressing the variability and random occurrences that are especially common in biomedical datasets. The architecture could identify both small and abstract anatomy through the addition of SE blocks and attention mechanisms. However, using this architecture in a non-biomedical application would likely be ineffective. For example, in autonomous vehicles when compared to current state-of-the-art models. This is because most non-biomedical datasets typically do not have the large amount of small variability that is present in biomedical datasets.

Similar to our proposed architecture, Schelmper et al. presented attention-gated networks for use in medical images. In their study, their network used 3D-CT abdominal images and 2D fetal ultrasound images, while obtaining results that outperformed the base U-Net architecture [19]. The higher DSC with lower variance is similar to the results that we obtained in our proposed CASE-Net architecture. Their work demonstrated that the attention-gated networks are able to exploit global and local object localization to improve model performance. The authors concluded that their attention-gated mechanisms perform well for tissue/organ identification and localization in fetal ultrasound. Having similar datasets in the same domain is what led to similar results between our proposed architectures and datasets.

Other fetal segmentation algorithms suffer from the same setbacks of fetal limb motion and position variability. For example, Zhang et al. presented a graph theory automated solution for full body fetal segmentation and obtained good results of only 12% error in their fetal body volume estimates. Nonetheless, their segmentation results were not provided, preventing more comprehensive evaluation of the accuracy. The algorithm in the study by Zhang et al. was only designed with 10 fetuses, while our algorithm was trained, validated, and tested using a total of 34 fetuses, improving our versatility and methodological rigor [2].

Many of the other segmentation tasks on whole fetal volumes or whole fetal envelopes are done with ultrasound images, which are much more limited because of the restrictions in field of view and resolution, resulting in lower segmentation accuracies ranging from 60% to 70% [18,23]. Ravishankar et al. were able to achieve an excellent average DSC of 90% when automatically segmenting the fetal abdomen in MRI. The slightly higher DSC in their study may be attributed to the exclusion of the limbs, hands, feet, and fingers, which typically show the highest amount of motion artifacts [24]. Other high-performing fetal segmentation algorithms were not fully automated [12]. The errors in our study could be attributed to the challenge of labeling the finer details of the fetal body. Although interactive segmentation algorithms have their own unique benefits, the completely automated approach is ideal for fast-paced, high-throughput applications. For example, automated algorithms are preferable in medical screening for anatomical abnormalities or health concerns. These segmentations and calculations can be done without the need for a medical professional, allowing important abnormalities detected by the algorithm to be flagged for more immediate review by a clinician.

## 5. Conclusions

Our study presents a novel automated whole fetal MRI segmentation method using DL architectures. CASE-Net is an architecture that combines the advantages of adaptive feature weights and attention gating to improve segmentation performance. Our network shows promising results in a difficult fetal segmentation task, in addition to outperforming other prominent DL architectures. The architecture was successful in fetal MRI segmentation, largely due to its superior localization of important contextual information.

## Figures and Tables

**Table 1:** MSE of the Dataset Compared with the Output Produced by the Algorithm.

	Dataset 1	Dataset 2	Dataset 3	Dataset 4	Dataset 5	Dataset 6
Person 1	495.54	366.36	1101.5	612.45	444.3	456.48
Person 2	465.54	309.99	1158.5	522.39	350.33	370.46

**Table 2:** Aggregated Mean Testing Results with Standard Deviation.

Model	Loss	DSC	Recall	Precision
CASE-Net	0.005	87.36 ± 0.83	91.79 ± 0.44	95.54 ± 0.13
UNet	0.069	85.17 ± 0.41	89.24 ± 0.26	94.55 ± 0.17
USE-Net	0.04	86.07 ± 0.41	86.61 ± 2.49	96.74 ± 0.27
ATN-Net	0.131	82.27 ± 0.47	88.69 ± 1.25	96.51 ± 0.27
Link-Net	0.096	82.72 ± 0.61	80.39 ± 4.96	95.86 ± 0.27

**Table 3:** CASE-Net Ablation Study with Standard Deviation.

Method	Loss	DSC	Recall	Precision
CASE-Net	0.005	87.36 ± 0.83	91.79 ± 0.44	95.54 ± 0.13
W/3 × 3 Kernel	0.052	83.50 ± 0.35	86.51 ± 2.35	96.47 ± 0.24
W/O ATN	0.018	84.35 ± 0.13	89.06 ± 1.78	96.26 ± 0.20
W/O SE	0.11	81.19 ± 1.20	80.95 ± 4.20	96.69 ± 0.40

## Data Availability

Not Applicable.
